# CREATIVE ACTIVITIES AND COGNITION AMONG OLDER ADULTS: DOES EDUCATION MODERATE THE BENEFITS OF CREATIVE ACTIVITIES?

**DOI:** 10.1093/geroni/igad104.3254

**Published:** 2023-12-21

**Authors:** Janelle Fassi

**Affiliations:** University of Massachusetts Boston, Boston, Massachusetts, United States

## Abstract

Engagement in leisure activities has been shown to offset age-related cognitive losses. Although activity engagement has a compensatory effect on later life cognition among adults with low educational levels in early life, no studies have examined this effect with creative activity engagement. The aims of the present study were to (1) investigate whether older adults’ engagement in any creative leisure activities is related to cognitive functioning, (2) investigate whether the number of creative activities in which older adults engage is related to cognitive functioning, and (3) determine whether education moderates the relationship between creative engagement and cognitive functioning. This present study utilized a nationally representative sample of 2,462 adults aged 65 years and older from the Health and Retirement Study, who completed either the 2016 or 2018 waves of the Leave Behind Questionnaire and the 2015 wave of the Consumption and Activities Mail Survey. Results from linear regression models revealed that creative engagement was associated with better cognitive functioning scores (β=0.74, p<.001). Additionally, more years of education were associated with better cognitive functioning scores (β=0.49, p<.001). Engaging in four creative activities is also associated with better cognitive functioning compared to engaging in no activities (β=1.38, p<.01). In sum, the results were in line with previous research that creative engagement and education are positively associated with cognitive functioning. Contrary to previous findings, creative engagement did not have a compensatory effect on cognitive functioning among individuals with fewer years of education.

